# Exploration of an urban lake management model to simulate chlorine interference based on the ecological relationships among aquatic species

**DOI:** 10.1038/s41598-018-26634-8

**Published:** 2018-05-29

**Authors:** Zhiqiang Yan, Yafei Wang, Di Wu, Beicheng Xia

**Affiliations:** School of Environmental Science and Engineering, Sun Yat-sen University, Guangzhou, 510275 China

## Abstract

In eutrophic lakes, algae are known to be sensitive to chlorine, but the impact of chlorine on the wider ecosystem has not been investigated. To quantitatively investigate the effects of chlorine on the urban lake ecosystem and analyze the changes in the aquatic ecosystem structure, a dynamic response model of aquatic species to chlorine was constructed based on the biomass density dynamics of aquatic species of submerged macrophytes, phytoplankton, zooplankton, periphyton, and benthos. The parameters were calibrated using data from the literature and two simulative experiments. The model was then validated using field data from an urban lake with a surface area of approximately 8000 m^2^ located in the downtown area of Guangzhou, South China. The correlation coefficient (R), root mean square error-observations standard deviation ratio (RSR) and index of agreement (IOA) were used to evaluate the accuracy and reliability of the model and the results were consistent with the observations (0.446 R < 0.985, RSR < 0.7, IOA > 0.6). Comparisons between the simulated and observed trends confirmed the feasibility of using this model to investigate the dynamics of aquatic species under chlorine interference. The model can help managers apply a modest amount of chlorine to control eutrophication and provides scientific support for the management of urban lakes.

## Introduction

Lake eutrophication is recognized to have deleterious effects on aquatic ecosystems, environmental systems and economies worldwide^1,2^. Eutrophication leads to harmful algal blooms and the formation of hypoxic environments due to high nutrient loads^3,4^. Since the 1960s, many studies, techniques, and policies for eutrophication control have been proposed and developed^5–7^. Reducing external nutrient sources and restoring normal ecological conditions are accepted as the main measures for controlling eutrophication^8,9^. However, due to the lack of a reliable, quantitative understanding of the ecological interactions between the internal and external environments of lake ecosystems, current urban lakes management is difficult to be performed specifically, thus remains “rough and wild”, particularly for the problem of lake eutrophication^10^. For example, the lack of reliable and quantitative interpretations of lake ecosystems has led to lake eutrophication dynamic that present a long duration and rebound effect. Therefore, quantitative models have been developed and are considered potential tools for eutrophication management^11,12^. These models are used to simulate the dynamics of each component in the lake ecosystem, which are complicated and include self-regulation and feedback^13^. Lake models can contribute to a better understanding of the ecological mechanisms in lake ecosystems and provide effective technical support for managers to evaluate environmental problems, predict environmental benefits and analyze environmental risks^14–17^. Lake models have been successfully developed in recent decades and expanded from monolayer, single ventricular components with zero dimensions to multi-level, multi-chamber components in three dimensions^18^. Among these models, integrated models including AQUATOX^19^, PAMOLARE^20^, CAEDYM^21^ and WASP^22^, have been established and applied in lake environment research and management. Despite the wide variety of available models, most focus on large lakes, whereas urban lakes, which play important and indispensable roles in urban water systems and microclimate regulation, receive much less attention^23^. Urban lakes, which provide both cultural and natural landscapes, act as important recreation sites and are closely related to citizens’ daily lives. Compared with lakes far away from cities, urban lakes are generally characterized as small, shallow and subject to frequent human impacts^24^. Urban lakes generate landscape heterogeneity and ecological diversity within a city. In addition, via the runoff, storage and infiltration of water, urban lakes can protect freshwater resources, control flooding, replenish groundwater and regulate the urban microclimate^25^. Despite their important functions in the urban ecosystem, urban lakes are easily affected or damaged by sewage discharge, storm runoff, and human activities^26,27^. Urban lake ecosystems are delicate and difficult to manage in a self-sustaining manner^28^. In recent years, urban lakes have increasingly suffered from a series of environmental problems, including the deterioration of water quality and, in particular, eutrophication events. For example, Lake Pamvotis in Ioannina, Greece, and Lake Kuminghu in Beijing, China, have experienced eutrophication problems at different levels^29,30^. To address eutrophication problems and prevent crashes in aquatic ecosystem balance, an important first step is to reveal the regulatory mechanisms of the lake ecosystem balance by constructing specific models.

Modeling studies of urban lakes are not being wide spread in the literature^31^. To date, most reports and discussions have concentrated on simulating hydrodynamics behaviors and water quality changes both physically and chemically but have not considered ecological processes^32^. Researchers have suggested establishing detailed models for shallow urban lakes to explore ecological heterogeneities and problems^33^. Lake ecosystems reflect the processes of exchanging material and energy with the atmosphere, water, sediment, and organisms. By now, most ecological models for lake ecosystems have focused on genera since the genera-specific model has many obvious merits, such as user-friendly, easy to calibrate and validate. However, many biological characteristics of species are unique. If the constructed model of aquatic populations is based on genera, the parameters might not be unique and stable, and possibly result in an insufficient understanding of the ecological functions of some important species^14,34^. Therefore, a species-specific model, that treats aquatic species as basic components and accurately simulates the dynamics of these species, is essential to complete and strengthen the urban lake management, and provide one more choice for studying.

Urban lake management should be based on a comprehensive understanding of the ecological dynamics of aquatic ecosystems^35^. The main tasks of lake management are to regulate the balance of the aquatic ecosystem and mitigate the impacts of external interferences^36^. Adding chlorine was proved to be an effective method to control the lake eutrophication, and thus has been applied for lake management^37,38^. Chlorine reacts with water to produce small molecules of hypochlorous acid. Hypochlorous acid spreads along the surface of the algal cell, penetrating the membrane and oxidizing intracellular protein, resulting in the inhibition of algae growth^39^. However, in lake management identifying effective measures for a specific lake is challenging due to the lack of a reliable quantitative understanding of the consequences of chlorine^40^. A low concentration of chlorine might not have a sufficient inhibitory effect on algae, whereas excessive chlorine can have devastating consequences on biological species. Therefore, using models to quantitatively simulate the effects of chlorine on aquatic species is necessary for environment managers and researchers to apply a modest amount of chlorine.

In this study, a dynamic model of a shallow urban lake was constructed based on the dynamics of primary aquatic species. The model considers individual aquatic species and chlorine interferences to simulate a shallow urban lake ecosystem. Under chlorine interference, the dynamics of several aquatic organisms and water quality were simulated with a specific model that integrates organism biomass dynamics, the ecological relationships between aquatic species, and phosphorus and chlorine in water. The model can help managers apply a modest amount of chlorine to control eutrophication and provides scientific support for the management of urban lakes. Furthermore, the structure and parameter values of this model can provide reference data for the study of urban lake models in the near areas.

## Results

### Calibration and validation

The calibrated parameters based on the results of the two experimental results or obtained from the literature are presented in Table 1. Model validation was performed using the monitored data from Lotus Lake.

**Table 1:** Model input parameters based on experiments or obtained from the literature, (the validation parameters are shown in brackets).

Species^#^ Symbol	a	b	c	d	e	f	g	h	i	j	k	l	m	n
Ae									0.3^50^	0.3^50^	0.3^50^	0.023	0.023	0.023
Iv	3423	0.34	2.13	0.52	2.01	6.16	2.05	17.11	0.000717	0.046159	0.25448	4.1425	46.3430	11.5858
Ib									1.45^50^	1.45^50^	1.45^50^	0.001^50^	0.001^50^	0.001^50^
Ik	2.2464^54^	2^49^	2^49^	2^49^	2^49^	0.09^54^	0.09^54^	0.09^54^						
Tk	1.1	1.1	1.1	1.1	1.1	1.1	1.1	1.1	1.05	1.05	1.05	1.05	1.05	1.05
Maxg	0.1^[e2]^	1.3^[e2]^	1.2^[e2]^	1.1^[e2]^	0.5~0.8(0.65)^[e2]^	0.065^[e2]^	0.065^[e2]^	0.065^[e2]^						
Maxm	0.03^[e2]^	0.007^[e2]^	0.007^[e2]^	0.007^[e2]^	0.007^[e2]^	0.005^[e2]^	0.005^[e2]^	0.005^[e2]^	0.14^49^	0.14^49^	0.14^49^	10^−4^ ^49^	10^−4 [b]^	10^−4[b]^
Maxr	0.022^[e2]^					0.0005^[e2]^	0.0005^[e2]^	0.0005^[e2]^	0.02^[e2]^	0.02^[e2]^	0.02^[e2]^	0.01^50^	0.026^50^	0.026^50^
Pk		0.03^53^	1^49^	1^49^	1^49^	1^49^	0.045^53^	0.045^53^	0.045^53^					
Rf									0.07^50^	0.07^50^	0.07^50^			
Sr		0.019^[e2]^	0.019^[e2]^	0.019^[e2]^	0.019^[e2]^									
Sk	0~0.00023^[e2]^ (0.0001)	0~0.1(0.06)^[e2]^	0.03~0.4(0.2)^[e2]^	0.35~0.55(0.4)^[e2]^	0.2~0.4(0.3)^[e2]^	0.001^[e2]^	0.001~0.05^[e2]^	0.003~0.06^[e2]^	0.0001^[e2]^	0.0001^[e2]^	0.0001^[e2]^			
Smk		0.0038^[e2]^	0.0038^[e2]^	0.003^[e2]^	0.003^[e2]^	0.00001^[e2]^	0.00001^[e2]^	0.00001^[e2]^						
Kcc		0.11~0.17(0.14)^52^	0.001^[e1]^	0.085~0.14(0.1)^52^	0.001^[e1]^	0.096^[e1]^	0.06~0.1(0.08)^[e1]^	0.1^[e1]^	0.05~0.54(0.3)^51^	0.15~0.76(0.3)^51^	0.3~0.81(0.6)^51^			

a Vallisneria natans (Lour.) Hara; b Microcystis aeruginosa; c Aphanizomenon flos-aquae; d Euglena gracilis; e Melosira granulata (Ehr.) Ralfs; f Ulothrix tenerrima (Kütz.) Kütz; g Oscill atoria chlorine; h Synedra acus; i Brachionus plicatilis; j Diaphanosoma brachyurum (Liévin); k Mesocyclops leuckarti (Claus); l Chironomid larvae; m Pomacea canaliculata (Caenogastropoda, Ampullariidae); n Gyraulus compressus(Hütton).

[e1] experiment 1; and [e2] experiment 2.

The attenuation model of chlorine according to the results of experiment 1 is presented in Table 2. The R values ranged between 0.9130 and 0.9601, reflecting good agreement between the model and the observations. The results of the regression analysis is showed the linear relationship between C0 and K. A higher initial concentration of chlorine corresponded to slower attenuation. The root mean square error-observations standard deviation ratio (RSR) was 0.55, which indicated good performance.

**Table 2:** Attenuation model of chlorine.

C_0_(mg L^−1^)	k	R	Equation
100	2.041	0.9130	C(t) = C_0_ * exp[−2.041 * (t-t_0_)]
150	0.9161	0.9357	C(t) = C_0_ * exp[−0.9161 * (t-t_0_)]
200	1.168	0.9601	C(t) = C_0_ * exp[−1.168 * (t-t_0_)]
250	0.5452	0.9421	C(t) = C_0_ * exp[−0.5452 * (t-t_0_)]
300	0.09466	0.9171	C(t) = C_0_ * exp[−0.09466 * (t-t_0_)]

Table 3 shows the results of the model evaluation for each component. The simulated values showed good qualitative agreement with the measurements based on the R, RSR and IOA measures. The R values ranged between 0.446 and 0.985. Except for the simulated biomass densities of *M*. *lopsleuckarti* (Claus) and *G*. *compressus* (Hütton), the simulated values presented highly significant correlations with the measurements (p < 0.01). In addition, the RSR values for all of the components were less than 0.7 and were thus satisfactory. The values of IOA for all the components were greater than 0.6, reflecting consistency between the model and observations.

**Table 3:** R, RSR, and IOA values indicating the agreement between the measured and simulated values.

Components	Correlation coefficient (R)	Root mean square error observations standard deviation ratio (RSR)	Index of agreement (IOA)
*V*. *natans* (Lour.) Hara	0.985**	0.09	0.872
*M*. *aeruginosa*	0.908**	0.431	0.951
*A*. *flos*-*aquae*	0.885**	0.697	0.941
*E*. *gracilis*	0.731**	0.692	0.838
*M*. *granulata* (Ehr.) Ralfs	0.931**	0.479	0.948
*U*.*tenerrima* (Kütz.) Kütz.	0.860**	0.582	0.893
*O*. *chlorine*	0.933**	0.004	0.936
*S*.*acus*	0.965**	0.017	0.976
*R*. *plicatilis*,	0.839**	0.604	0.998
*D*. *brachyurum* Liévin	0.940**	0.374	0.967
*M*.*leuckarti* (Claus)	0.446*	0.231	0.668
Chironomid larvae	0.902**	0.591	0.932
*P*.*canaliculata*(*Caenogastropoda*, *Ampullariidae*)	0.820**	0.518	0.848
*G*.*compressus*(Hütton)	0.763*	0.496	0.749
TP in water	0.967**	0.257	0.898
TP in sediment	0.875**	0.607	0.983

**Significant at p < 0.01, *significant at p < 0.05, 0 < RSR < 0.5 indicates very good performance, 0.5 < RSR < 0.6 indicates good performance, 0.6 < RSR < 0.7 indicates satisfactory performance, and 0.7 > RSR indicates unsatisfactory performance^61^.

### Simulation of TP in water and sediment

**Figure 1:**
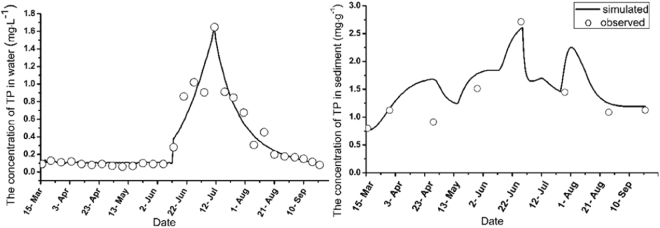
The observed and simulated values of TP in water and sediment.

Figure 1 shows the total phosphorus concentrations according to the modeled data and the field data for lake water and sediment.

The TP in water presented a single peak (Fig. 1(a)) with a value of approximately 1.65 mg L^−1^. Although differences were observed between the measurement and simulation values, the trends of TP concentration obtained from the model were generally in agreement with the observations. The increase in TP not only led to the outbreak of eutrophication but also resulted in an increase of TP in sediment (Fig. 1(b)) due to settling during this period.

### V. natans (Lour.) Hara

**Figure 2:**
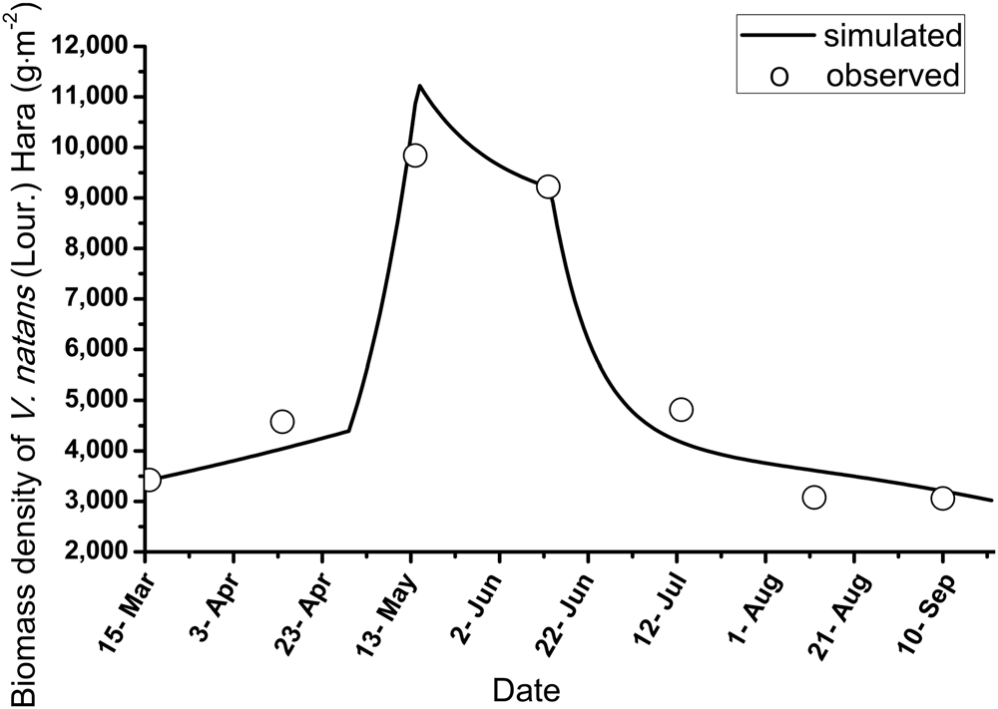
Observed and simulated values of the biomass density of V. natans (Lour.) Hara.

The simulated and observed biomass densities of *V*. *natans* (Lour.) Hara are shown in Fig. 2.

**Figure 3:**
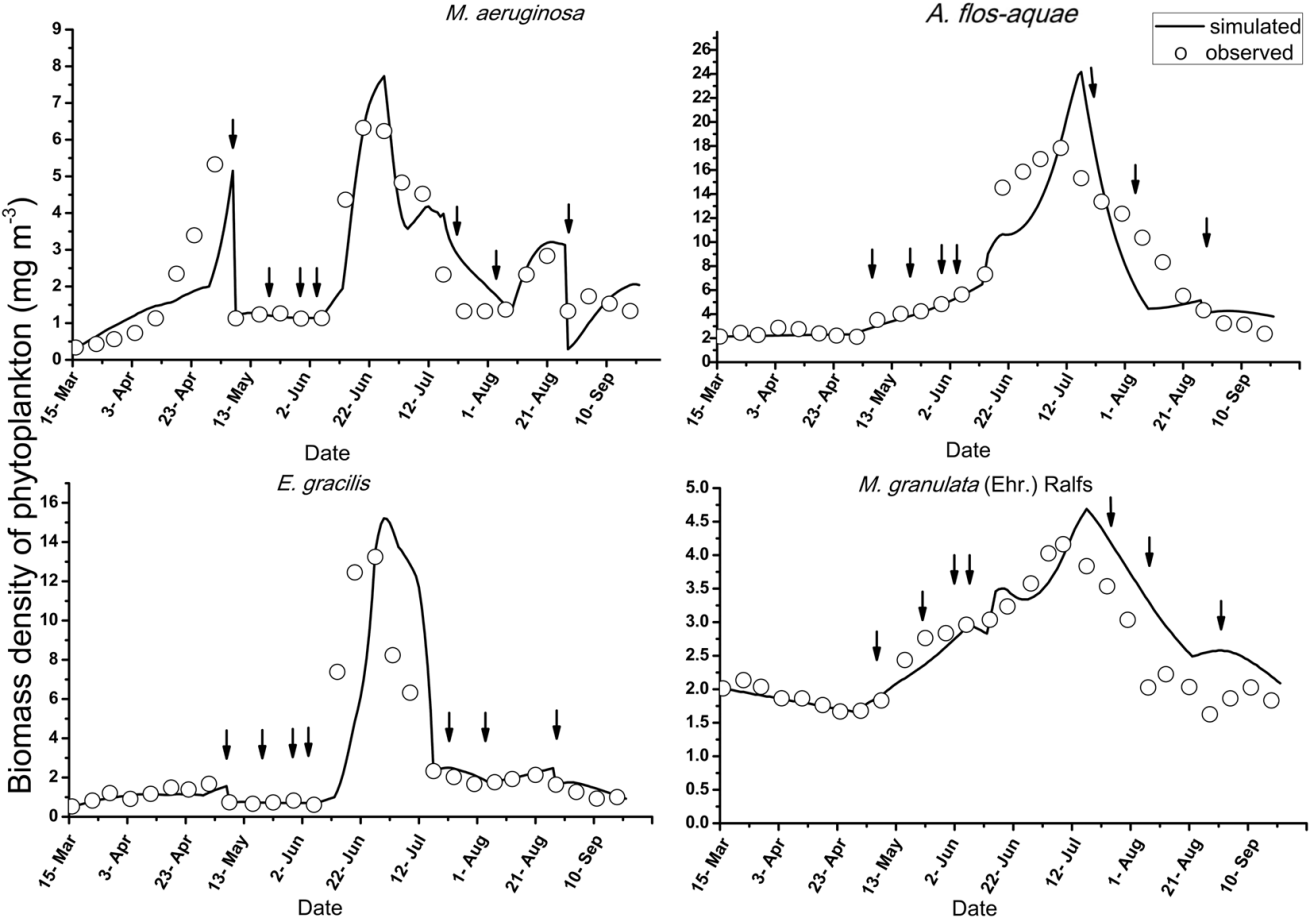
Observed and simulated biomass density values of the four main species of phytoplankton in the lake (the arrows indicate the chlorine interference).

**Figure 4:**
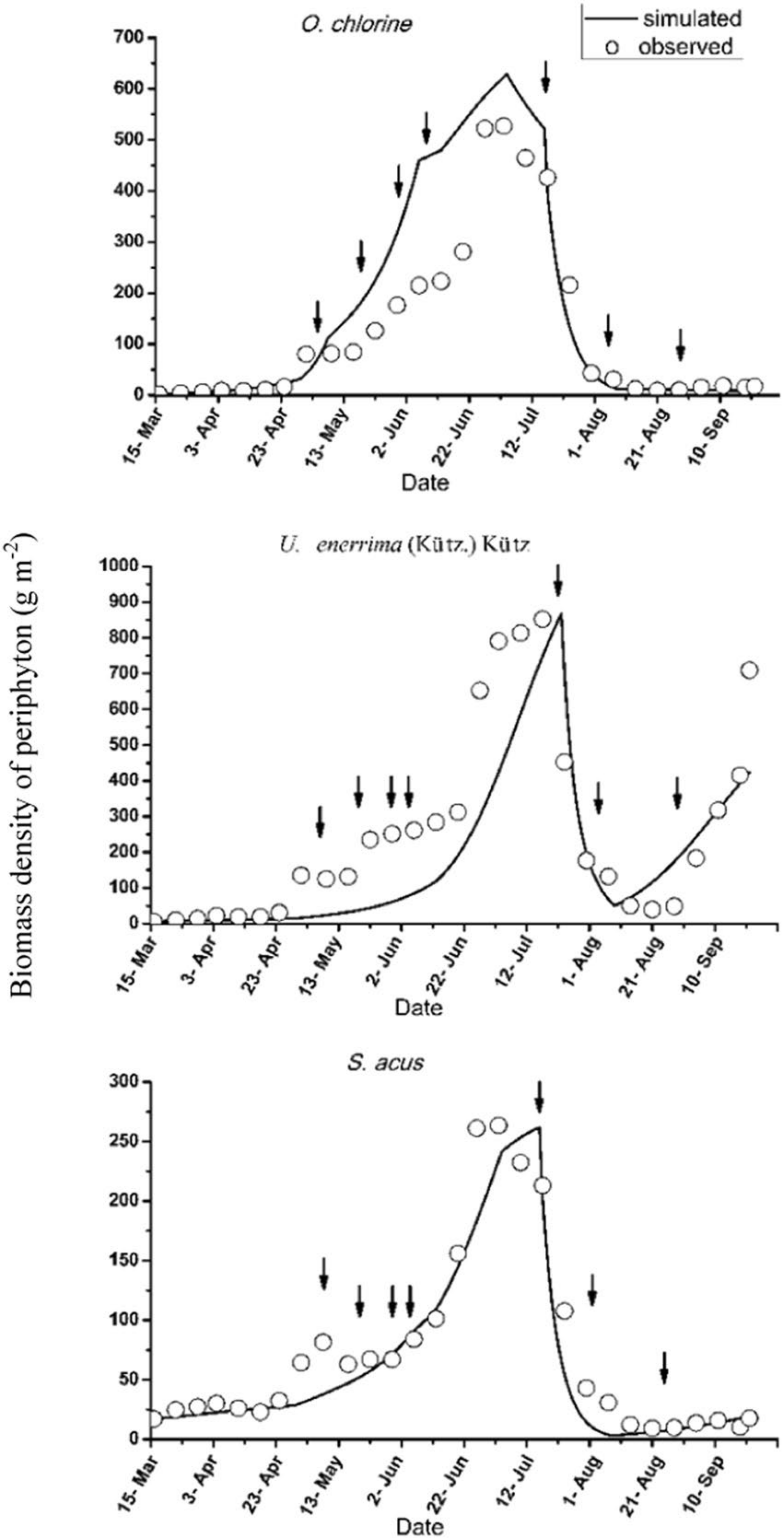
Observed and simulated biomass density values of three main kinds of periphyton in the lake (the arrows represent chlorine interference).

A single peak was found for the biomass density of *V*. *natans* (Lour.) Hara. The simulated values were analogous to the observed values, with corresponding peaks in May. Although the simulated maximum values were larger than the observed values, the relative error was less than 15% (12.3%). The simulated results were reasonable. However, eutrophication began in the lake on May 10^th^, resulting in the decline of *V*. *natans* (Lour.) Hara biomass density. Both the observations and simulations followed decreasing trends after mid-June, when phytoplankton and periphyton appeared to be blooming (Figs 3 and 4). Subsequently, the biomass density of *V*. *natans* (Lour.) Hara continued to decrease, reaching a measured density of approximately 3000 g·m^−2^ in September.

### Phytoplankton

The main species of phytoplankton in the lake were *M*. *aeruginosa*, *A*. *flos*-*aquae*, *E*. *gracilis*, and *M*. *granulata* (Ehr.) Ralfs. The simulated and observed biomass densities of the four main species of phytoplankton are presented in Fig. 3.

Three peaks were found in the biomass density dynamics of *M*. *aeruginosa*, whereas only one peak was found for each of the remaining three species. The simulated and observed patterns of the densities of the four phytoplankton species presented similar trends. *M*. *aeruginosa* was clearly very sensitive to chlorine. Decreases in the biomass density of *M*. *aeruginosa* and *E*. *gracilis* were observed on May 8^th^, 19^th^, and 29^th^, June 4^th^, July 17^th^ and August 4^th^ and August 28^th^ based on both the observations and simulations due to the intense inhibitory effects of chlorine. In addition, both the simulated and observed values for *A*. *flos*-*aquae* and *M*. *granulata* (Ehr.) Ralfs. presented increasing trends after the application of chlorine on May 8^th^, 19^th^, and 29^th^and June 4^th^, reflecting a weak effect of chlorine on these species. The peaks in density for four species of phytoplankton presented distinct delays in the simulations relative to the observed values. Although the modeled values agreed well with the observations, the observed maximum values were smaller than the corresponding simulated values.

### Periphyton

*O*. *chlorine*, *U*. *tenerrima* (*Kütz*.) *Kütz*, and *S*. *acus* were the dominant species of periphyton in the lake. Figure 4 shows the simulated and observed biomass density values for these three main species.

Unlike the biomass densities of phytoplankton, the biomass densities of periphyton displayed a single peak according to both observation and simulation. Although differences were observed between the observations and simulations, the trends and density values obtained from the model were generally consistent with the observations. In addition, the simulated maximum biomass densities of each of the three species of periphyton agreed well with the observations; however, the observed duration of the *O*. *chlorine* bloom was shorter than the simulated duration, whereas the opposite pattern was observed for *U*. *tenerrima* (Kütz.) Kütz. Among the three species, *U*. *tenerrima* (Kütz.) Kütz. had the strongest restoration ability and exhibited an increasing trend after August 21^st^.

### Zooplankton and Benthos

**Figure 5:**
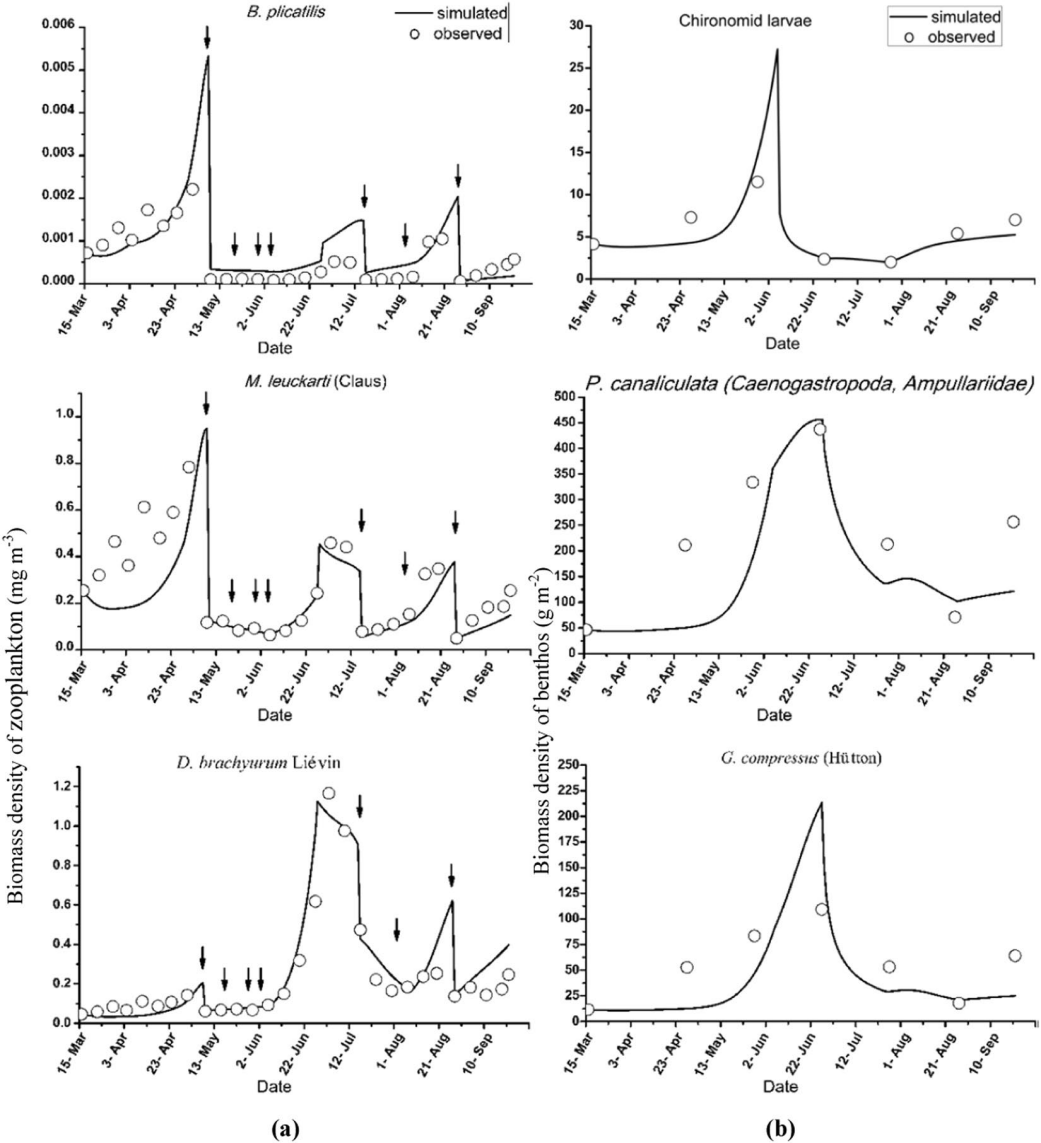
Observed and simulated biomass density values of three main species of zooplankton and benthos in the lake (the arrows represent chlorine interference).

The main species of zooplankton in the lake were *B*. *plicatilis*, *M*. *leuckarti* (Claus) and *D*. *brachyurum* (Liévin). Chironomid larvae, *P*. *canaliculata* (*Caenogastropoda* and *Ampullariidae*) and *G*. *compressus* (Hütton) were the dominant species of benthos in the lake. The simulated and observed biomass density values of the three main species of each zooplankton and benthos are presented in Fig. 5.

Three peaks were observed in the biomass density of all three main species of zooplankton (Fig. 5(a)), with similar values observed between the simulations and observations. A decrease in the biomass density of each of the three species occurred when chlorine was applied on May 8^th^, 19^th^, and 29^th^, June 4^th^, July 17^th^, and August 4^th^ and August 28^th^. The modeled peaks of *B*. *plicatilis* were obviously higher than the corresponding observed values, and this pattern was also observed in the other two zooplankton species, but the discrepancies were not obvious. Despite that, the relative error between the simulations and observations was relatively small. A single peak in the biomass density of each of the benthos species was observed (Fig. 5(b)). Although differences were observed between the measurements and simulations, the trends and values obtained from the model were generally in agreement with the observations. The modeled peaks for the Chironomid larvae and *G*. *compressus* (Hütton) were much higher than the observed peaks.

Overall, this study explored the responses of the aquatic species to chlorine interference with two initial concentrations, i.e. 0.045 and 0.188 mg/L. The results showed that, within this concentration range, chlorine has a remarkable inhibitory effect on the dominant species (*M*. *aeruginosa*), thus control eutrophication in the urban lake.

## Discussion

The model developed in this study coupled dynamic modules of biomass density of the main aquatic species with environmental factors. Table 4 lists the similar models from other studies^41–43^. Most of these models focused on the dynamic simulation of nutrients and phytoplankton or chlorophyll-a. Marchi developed a model to simulate the seasonal dynamics of major components of Lake Chozas, when an invasive fish was introduced into the lake. Compared with the previous studies, the model developed in this study focused on species instead of genera. Addressing only the dynamics of a category of aquatic organisms, obviously, ignores the ecological functions of certain important species. Although a simpler genera-specific model may be easier to use, calibrate and validate, a species-specific model based on the dynamics of primary aquatic species provides a tool for the researchers and managers who aim to explore the dynamic of species and to understand the mechanisms among aquatic species. Therefore, the constructed model in this study is essential to help the manager to complete and strengthen the urban lake management.

**Table 4:** Models published in the literatures.

Object	State Variables	Functions/Equations	References
Yuqiao Reservoir	Epi Sm	7	^12^
Chozas Lake	Phy Zoo TN TP Det Psed Fish	30	^49^
Chesapeake Bay	TP TN SS DO Zoo Chla Sm Ben	7	^50^
Washington Lake	Phy Zoo OC TN TP SiO_2_ DO	59	^41^
Taihu Lake	Phy Zoo TN TP Det DOM DO Psed Nsed Csed	19	^42^
Dianchi Lake	Chla TN TP NH_4_ NO_3_ ON	13	^43^

Epi, epiphyton; Sm, submerged macrophytes; Phy, phytoplankton; Zoo, zooplankton; TN, total nitrogen in water; TP, total phosphorus in water; Det, detritus; Psed, total phosphorus in sediment; SS, suspended solid; DO, dissolved oxygen in water; Chla, chlorophyll-a; Ben, benthos; OC, organic carbon; DOM, dissolved organic matter; Nsed, total nitrogen in sediment; Csed, carbon in sediment; NH_4_, ammoniacal nitrogen in water; NO_3_, nitrate nitrogen; ON, organic nitrogen.

In addition, using chlorine is an effective method to manage the lake eutrophication, hence it is necessary to comprehensively understand the ecological dynamics of aquatic ecosystems under chlorine interference in urban lake management. This model, using the exponential function to couple the chlorine interference and accurately simulating the responses of the species to chlorine interference, can help managers apply a modest amount of chlorine to control eutrophication and provides scientific support for the management of urban lakes.

The dynamic model presented in this study explained the radical changes observed in the shallow urban lake. Both the simulated and observed results showed that the algae with cystic structure and zooplankton were sensitive to chlorine, whereas the algae with filamentous structure and submerged macrophytes showed strong resistance to chlorine. The increase in TP in the water between June 12^th^ and 22^nd^ clearly promoted the growth of phytoplankton and periphyton, which resulted in the breakout of eutrophication. The results confirmed that abundant TP nutrients provided the foundation for the phytoplankton outbreak. However, the TP additions were necessary but not sufficient^44^, as evidenced by the low TP concentrations between May 2^nd^ and June 12^th^. However, the biomass of *A*. *flos*-*aquae* and *M*. *granulata* (Ehr.) Ralfs presented increasing trends in the measured experiment. The biomass densities of phytoplankton and periphyton species presented decreasing trends from July 12^th^ to August 10^th^, resulting in a regime shift in the lake ecosystem from a turbid phase to a clean phase. During this period, the TP in water showed a decreasing trend (Fig. 1), thereby limiting the growth of phytoplankton and periphyton. Moreover, the intense interference of chlorine inhibited the growth of *M*. *aeruginosa*, which was an active species on July 17^th^ and August 4^th^.

By comparing the trends and biomass density values of the main aquatic species under the chlorine interferences between the simulations and observations, we found that the modeled results were acceptable with certain discrepancies. Chlorine can decay in a few hours. The attenuation model of chlorine ran with a time interval of one hour. However, the daily amount of chlorine in water used as input in the model was calculated using the exponential equation to produce a daily average. Thus, the calculation method and the time interval of chlorine concentrations might have introduced errors into the simulations. In addition, the density peaks of the four species of phytoplankton presented distinct delays in the simulations relative to the observed patterns, which was likely because the simulations were run without considering the hydrodynamic effects. Studies have found that small-scale motion or low flow velocity were generally favorable for algal growth and aggregation in monoculture or mixed culture^45^. Despite these discrepancies between the simulated and observed values, good agreement was observed for both the values and trends, and the model satisfactorily described the dynamics of aquatic species under chlorine interference. The structure and parameter values of this model can provide a reference for the study of urban lake models in the near area.

The model accurately simulated the dynamics of aquatic species with the chlorine interference in an urban lake. Differences between the observed and simulated values were inevitable because some important components of the ecosystem were ignored in the model, including fishes and bacteria species and hydrodynamic influences^46,47^. In addition, because of the limitations of study time and location, the further validation of the model on different lakes and other seasons are recommended. Nonetheless, the model incorporated the main species in each class of aquatic organism that performed critical functions in the lake ecosystem and exhibited good performance. Therefore, this study provides strong support for the use of the model for urban lake eutrophication management.

The application of constructed model should be based on the construction of new ecosystem. To extend this model to other similar urban lake ecosystems, the following steps are needed: (1) conducting ecological investigation of the lake, (2) identifying the dominant species, (3) calibrating the relevant parameters in the model.

## Conclusions

By now, urban lakes management remains rough, especially for the problem of lake eutrophication. The associated management measures often lack scientific support and are thus not effective and sufficiently. Here, an urban lake management model was developed, and the model provides scientific support for the management of urban lakes.

For the management and control of eutrophication problem, a comprehensive understanding of the ecological dynamics of aquatic ecosystems must be developed. A simulative model of aquatic ecosystem dynamics could assist managers in understanding the processes of eutrophication. In this study, a dynamic model of a shallow urban lake was constructed based on the biomass density of the aquatic species subjected to frequent and strong chlorine interference. The test results showed that the simulation was highly accurate and consistent with field variations. Thus, this model is appropriate for addressing urban lake eutrophication.

The effect of chlorine on different aquatic species was studied and found to help regulate the balance of aquatic species, and the model simulation could reveal the direction of the balance of the aquatic ecosystem. The model developed in this study can provide guidance on the interactions between aquatic species growth and chlorine applying. The consistency between the field investigation and model outputs provides strong support for the use of the model for urban lake eutrophication management, including the application time and chlorine dosages based on aquatic organism dynamics.

The model was established via laboratory tests, experiments, field investigation and previous literature, and the statistical tests showed appropriate results. The reliability of the model also provides support for the management of urban lakes and shows that simple and effective chemical methods, such as the application of chlorine, should be considered. The structure and parameter values of this model can provide a reference for the study of urban lake models in the near area.

Further refinements of urban lake eutrophication management should be coupled with the use of bio-regulators.

## Materials and Methods

### Dynamic model of biomass density

**Figure 6:**
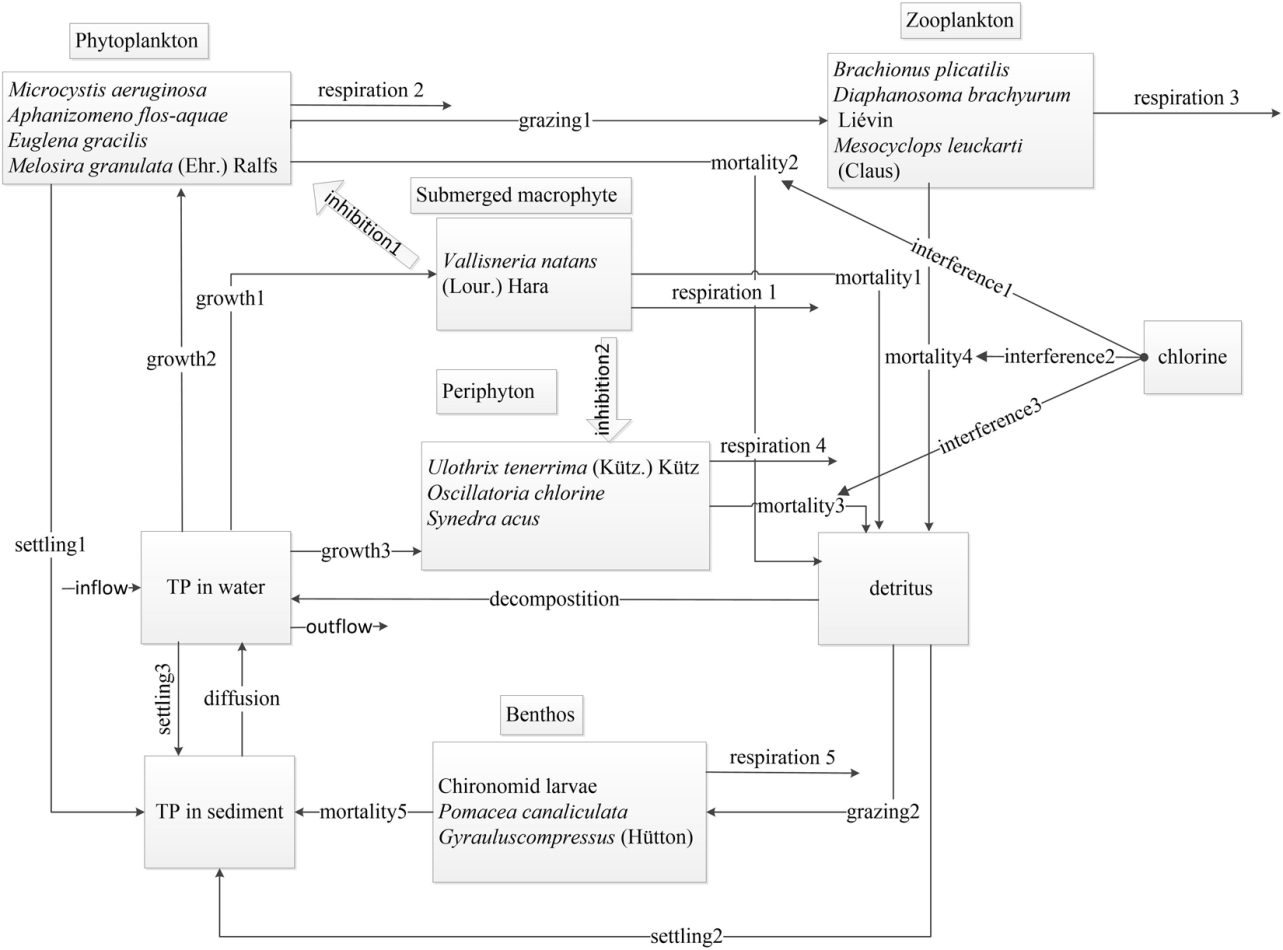
Conceptual diagram of the model (the arrows represent kinetic interactions among components).

A simulation model for the lake is developed by coupling the biomass density dynamic modules of the main aquatic organisms and environmental factors. The model is developed using Stella software (version9.1.4). The system dynamics of the model presents a strong modeling environment and a simple operation mode^48^. The model structure is presented in Fig. 6. *Vallisneria natans* (Lour.) Hara, phytoplankton, zooplankton, periphyton, benthos, detritus and the total phosphorus in water and sediment are considered the main components of the model. The phosphorus in water, as a linkage in the system, is directly related to the growth of submerged macrophytes, phytoplankton and periphyton and to the sediment of the lake. The food chain, competition, predation, respiration, and mortality are the main processes in the model. Chlorine is the only artificial intervention included in the system. Tables 5 and 6 show the different parameters and equations in the model.

**Table 5:** Summary of the symbols in the model.

Symbol	Description	Unit
Ae	Assimilation efficiency of zooplankton	day^−1^
As	Active layer of sediment	m
Biom(i)	Biomass density	
Biomphy	Biomass density of phytoplankton species	
Biomsvm	Biomass density of *V*. *natans* (*Lour*.) Hara	
Biomzoo	Biomass density of zooplankton species	
Cc	Concentration of chlorine	mg L^−1^
D	Depth of the lake.	m
Dec	Decomposition biomass rate of detritus	day^−1^
Det	Biomass density of detritus	
Diff	Diffusion of TP from the sediment	
Dr	Decomposition rate of detritus	day^−1^
Drr	Diffusion rate of phosphorus	day^−1^
Grazing(i)	Biomass of the phytoplankton grazed upon by zooplankton	
Grazing2	Biomass density of detritus grazed upon by benthos	
Growth(i)	Process of growth	
I	Species	
Ib	Ingestion rate of zooplankton	day^−1^
Ik	Half saturation constant for solar radiance	MJ m^−2^ day^−1^
Ip	Half saturation constant for phosphorus uptake from water	mg m^−3^ day^−1^
Tk	Temperature effect constant of species	
Tkp	Temperature effect constant for primary producers	
Kcc	Attenuation coefficients due to chlorine	day^−1^
Kpz	Half saturation constant of phytoplankton grazing for zooplankton	
Maxg	Maximum growth rates	day^−1^
Maxm	Maximum mortality rates	day^−1^
Maxr	Maximum respiration rates	day^−1^
Mortality(i)	Process of mortality	
Mortality1−5	Mortality of *V*. *natans* (*Lour*.) Hara, phytoplankton, periphyton, zooplankton and benthos	
Pw	Total phosphorus concentration in water	mg L^−1^
Ps	Total phosphorus concentration in sediment	mg g^−1^
Respiration(i)	Process of respiration	
Rf	Respiratory cost for grazing by zooplankton	day^−1^
Settling1	Settlement of phytoplankton	
Settling2	Settlement of detritus	
Settling3	Settlement of TP in water	
Sr	Solar radiance	MJ m^−2^ day^−1^
Sk	Attenuation coefficient due to self-population density	day^−1^
Smk	Attenuation coefficients due to *V*. *natans* (*Lour*.) Hara	day^−1^
Ser	Settling rate of phytoplankton	day^−1^
Serd	Settling rate of detritus	day^−1^
Serp	Settling rate of phosphorus in water	day^−1^
T	Temperature	°C

**Table 6:** Summary of the equations in the model.

Equations		Reference
Biom(i) = growth(i) − mortality(i) − respiration(i)	(1)	^50^
Biom(i) = growth(i) − mortality(i) − respiration(i) − settling(i) − grazing(i)	(2)	
$$\begin{array}{c}{\rm{Growth}}({\rm{i}})={\rm{Maxg}}({\rm{i}})\ast {\rm{Biom}}({\rm{i}})\ast (\frac{{\rm{pw}}}{{\rm{pw}}+{\rm{ip}}({\rm{i}})})\ast {\rm{Tkp}}\wedge ({\rm{T}}-20)\ast (\frac{{\rm{sr}}}{{\rm{sr}}+{\rm{ik}}({\rm{i}})})\\ \times \ast \exp (-{\rm{sk}}\ast {\rm{Biom}}({\rm{i}}))\end{array}$$	(3)	
$$\begin{array}{c}{\rm{Growth}}({\rm{i}})={\rm{Maxg}}({\rm{i}})\ast {\rm{Biom}}({\rm{i}})\ast (\frac{{\rm{pw}}}{{\rm{pw}}+{\rm{ip}}({\rm{i}})})\ast {\rm{Tk}}\wedge ({\rm{T}}-20)\ast (\frac{{\rm{sr}}}{{\rm{sr}}+{\rm{ik}}({\rm{i}})})\\ \times \ast \exp (-{\rm{sk}}\ast {\rm{Biom}}({\rm{i}}))\ast \exp (-{\rm{smk}}\ast {\rm{Biomsvm}})\end{array}$$	(4)	
$${\rm{Mortality}}({\rm{i}})={\rm{Biom}}({\rm{i}})\ast {\rm{Maxm}}({\rm{i}})\ast {\rm{Tk}}\wedge ({\rm{T}}-20)\ast \exp ({\rm{kcc}}\ast {\rm{cc}}\ast 1000)$$	(5)	
Respiration(i) = Tk^(T−20) * Maxr(i) * Biom(i)	(6)	
$$\begin{array}{c}{\rm{Grazing}}({\rm{i}})={\rm{Biomzoo}}({\rm{i}})\ast {\rm{ae}}({\rm{i}})\ast \text{ib}({\rm{i}})\ast ({\rm{Tk}}\wedge ({\rm{T}}-20))\ast (1-{\rm{rf}})\ast \frac{{\rm{Biomphy}}({\rm{i}})-0.5}{{\rm{Biomphy}}({\rm{i}})+{\rm{kpz}}({\rm{i}})}\\ \times \ast \exp (-{\rm{sk}}\ast {\rm{Biomzoo}}({\rm{i}}))\end{array}$$	(7)	
${\rm{Settling}}1=\frac{{\rm{ser}}({\rm{i}})}{{\rm{d}}}\ast (\frac{{\rm{T}}}{20})\ast {\rm{Biom}}({\rm{i}})$	(8)	^49^
Biom(i) = grazing(i) − mortality(i) − respiration(i)	(9)	
Grazing(i) = ae(i) * ib(i) * Tk^(T−20) * Biom(i) * det	(10)	
Mortality(i) = Tk^(T−20) * Maxm(i) * Biom(i) * exp(kcc * cc * 100)	(11)	
Respiration(i) = Biom(i) * Maxr(i) * Tk^(T−20)	(12)	
Det = mortality1 + mortality2 + mortality3 + mortality4 − settling2 − grazing2 − dec	(13)	
${\rm{Settling}}2=\,\frac{{\rm{serd}}}{{\rm{d}}}\ast (\frac{{\rm{T}}}{20})\ast {\rm{\det }}$	(14)	^49^
Dec = dr * det * Tk^(T − 20)	(15)	^49^
Pw = diff + dec − growth1 − growth2 − growth3 − settling3	(16)	
Ps = settling1 * 0.002 + settling2 * 0.002 + setting3 + mortality5 * 0.0001 − diff	(17)	
${\rm{Diff}}={\rm{drr}}\ast ({\rm{ps}}-{\rm{pw}})\ast \frac{{\rm{as}}}{{\rm{d}}}$	(18)	^49^
${\rm{Setting}}3=\frac{{\rm{serp}}}{{\rm{d}}}\,\ast \,\text{pw}\,$	(19)	^49^

### Primary producers

Submerged macrophytes, phytoplankton and periphyton are primary producers in the ecosystem and have similar ecological processes, including growth, death and respiration. The biomass density model of the submerged macrophytes, phytoplankton and periphyton is built based on the concepts of Marchi^49^ (equation 1). The model of phytoplankton also incorporates the settlement process and grazing process of zooplankton (equation 2). Growth is a function of temperature, phosphorus, light and population inhibition (equation 3). The growth of phytoplankton and periphyton also incorporates the inhibition of *V*. *natans* (Lour.) Hara growth (equation 4). Mortality is a function of temperature and chlorine (equation 5). Respiration loss is a function of temperature (equation 6). The grazing of zooplankton on phytoplankton is a function of temperature as well as phytoplankton and zooplankton populations (equation 7). The settling of phytoplankton is an exponential function and a function of the temperature and depth of the lake (equation 8). Key parameters for different species of primary producers are presented in Table 1.

### Consumers

The biomass density model of zooplankton and benthos is constructed based on the concepts established by Cerco^50^. Zooplankton and benthos are the main consumers in the model. Grazing, death and respiration are recognized as the main processes (equation 9). The grazing of zooplankton is a function of the temperature, assimilation efficiency, ingestion rate and phytoplankton (see equation 7), whereas the grazing of benthos is a function of temperature, assimilation efficiency, ingestion rate and detritus (equation 10).

### Detritus

Benthos grazing, decomposition and settling are recognized as the main processes for detritus (equation 13). The majority of the detritus originate from the mortality of biological components. The settling of detritus is a function of lake depth, the settling rate of detritus, the biomass density of detritus in water and temperature (equation 14). The decomposition of detritus is a function of temperature, the decomposition rate of detritus and the biomass density of detritus (equation 15).

### Total phosphorus (TP) in water and sediment

The uptake of primary producers, diffusion from sediment, decomposition of detritus, and settling are recognized as the main processes underlying TP variations in water (equation 16). The settling of TP in water, phytoplankton, detritus, decomposition of benthos and diffusion are the main processes for TP in sediment (equation 17). Diffusion is a function of the TP concentration in water and sediment, the diffusion rate of phosphorus, the lake depth and the active layer of sediment (equation 18). The settling of TP in water is a function of the lake depth, the settling rate of phosphorus and the TP concentration in water (equation 19).

### Parameter calibration

Calibration data are obtained from the literature and two experiments in the laboratory. Data on the ingestion rate, the assimilation efficiencies of zooplankton and benthos, and the respiratory cost for grazing by zooplankton are obtained from the literature^51^. The inhibitory rate of chlorine on phytoplankton and zooplankton is obtained from the literature^52,53^. The half saturation constants for phosphorus uptake from water (mg m^−3^ day^−1^) and the half saturation constant for solar radiance are obtained from the literature^54^. The attenuation rate of chlorine and the inhibitory rate of chlorine to submerged macrophytes are obtained from the data from experiment 1. The daily maximum growth, mortality and respiration rates of aquatic species, and the attenuation coefficient caused by the self-population density, as well as the attenuation coefficients of phytoplankton and periphyton due to *V*. *natans* (Lour.) Hara, are obtained from data in experiment 2.

#### Experiment 1

To explore the inhibitory rates of chlorine to filamentous algae and submerged macrophytes, a co-culture experiment is performed with *Spirogyra*.spp. and *V*. *natans* (Lour.) Hara at various initial concentrations of chlorine solution in the laboratory. The fresh weight of *Spirogyra*.spp. and *V*. *natans* (Lour.) Hara is 10 g and 15 g, respectively, and the organisms are cultured in a container (18.0 × 12.0 × 6.5 cm) containing 750 mL of chlorine solution. The initial concentration of chlorine solution, obtained using sodium hypochlorite solution, is set to 100, 150, 200, 250 or 300 mgL^−1^. Each treatment has three replicates. The chlorophyll-a contents of *Spirogyra*.spp. and *V*. *natans* (Lour.) Hara are measured at 24, 48, 72 and 96 h according to the acetone methods^55^. The concentration of chlorine is detected at 1, 2, 3, 6, 12, 18, 24, 48, 72 and 96 h according to standard methods^56^.

To understand the dependence between the initial concentration of chlorine solution (C_0_) and attenuation rate (k), a regression analysis is used. In addition, the root mean square error-observations standard deviation ratio (RSR, equation 23) is used to evaluate the accuracy of the curve estimation in the regression analysis.

#### Experiment 2

To explore the model parameters of primary producers and consumers, the effects of submerged macrophytes and daphnia magna on lake eutrophication are investigated in the laboratory. White polyethylene barrels with a volume of 65 L and a bottom sediment layer of 8 cm are used as the experimental containers. Fifty liters of water with 0.2 mg L^−1^ TP is transferred into each container. Next, *Microcystis aeruginosa*, *Aphanizomenon flos*-*aquae*, and *Melosira granulata* (Ehr.) Ralfsat a cell density of 3 * 10^6^ ind L^−1^ are transferred into each container to produce a micro-scale eutrophic shallow lake ecosystem. *M*. *aeruginosa*, *A*. *flos*-*aquae*, *M*. *granulata* (Ehr.) Ralfs and daphnia magna are obtained from the Institute of Hydrobiology, Chinese Academy Sciences, Wu Han, China. *Vallisneria natans* (Lour.) Hara is planted at a biomass density of 800 g m^−2^ to create one experimental group. The same density of *V*. *natans* (Lour.) Hara along with the large *Daphnia magna* at a density of 100 ind L^−1^ are used to produce a second experimental group. In addition, a controlling group is established. Each group has three replicates. The concentration of total phosphorus in water and the cell densities of the three types of algae and large daphnia are monitored daily. The biomass density of *V*. *natans* (Lour.) Hara and the concentration of total phosphorus in sediment are measured every 10 days. The biomass of *V*. *natans* (Lour.) Hara is measured at the end of the experiment. The period of the experiment spans 80 days. Species identification and biomass density measurements for the submerged macrophytes, phytoplankton, zooplankton, periphyton, and benthos are performed according to the standards for the investigation of reservoir fishery resources in China^57^. The total phosphorus of the water and sediment is detected according to standard methods^58,59^.

A sensitivity analysis of parameters is performed, and the basic steps are as follows: first, determine the ranges of parameter values according to the calibration results; second, input the maximum and minimum parameter values and the mean values input for the other parameters into the scenario; and finally, assessing the performance of the specified parameter via the mean relative error:

20 $${\rm{MRE}}=({\sum }_{{\rm{i}}=1}^{{\rm{n}}}|\frac{{\rm{Pmax}}-{\rm{Pmin}}}{{\rm{Pmax}}}|)/{\rm{n}}$$

where n is the length of the time series and Pmax and Pmin are the modeled maximum and minimum species concentration, respectively. The optimal values of the calibrated parameters obtained after the calibration are listed in the Table 1. The four most sensitive parameters listed in sequence according to the MRE values (Sk(*M*. *granulata* (*Ehr*.) Ralfs) = 85.5%; Sk(*A*. *flos*-*aquae*) = 51.1%; Sk(*V*. *natans* (*Lour*.) Hara) = 48.1% and Maxg(*M*. *granulata* (*Ehr*.) Ralfs) = 29.4%), are selected for calibration for the model.

### Model validation

Lotus Lake, located in the downtown area of Guangzhou (23°7′54″ N,113°15′42″ E), is a small and shallow lake with a surface area of approximately 8000 m^2^ and an average depth of 0.8 m. Guangzhou mainly has a classic marine subtropical monsoon climate, with an annual mean temperature of 21.5 °C and average rainfall of approximately 1736 mm. The submerged macrophyte, *V*. *natans* (Lour.) Hara is planted in January 2015 and covers the whole lake bottom by March 2015.

The study period of the field investigation is from March 15^th^ to September 30^th^, 2015. The surface shape of the lake is rectangular and divided into four parts by two intersecting diagonals. Samples of water, phytoplankton, zooplankton and periphyton are collected weekly, and sediment, submerged macrophyte and benthos samples are collected monthly at five points: one point at the center of the lakes and four other pointy, each in the center of one of the four parts. All samples are held at 4 °C in an insulating container and were taken back to the lab immediately.

Eutrophication in the lake appears repeatedly after May 10^th^, 2015. Exogenous pollutants are input into the lake on June 10^th^, resulting in an outbreak of eutrophication. To control eutrophication, 5000 g of calcium hypochlorite containing 30% chlorine (i.e. 0.188 mg/L chlorine), is applied to the lake on May 8^th^, 19^th^ and 29^th^; June 4^th^; July 17^th^; and August 4^th^; and 1200 g of calcium hypochlorite containing 30% chlorine (i.e. 0.045 mg/L chlorine) is applied on August 28^th^. The daily concentration of chlorine in the water is calculated using the exponential equation and then averaged over one hour.

21 $${\rm{Ci}}={\rm{Co}}\ast \exp ({\rm{k}}\ast {\rm{t}})$$

22 $$\bar{{\rm{C}}}=\frac{1}{24}\ast \sum _{{\rm{i}}=1}^{24}{\rm{Ci}}$$

where Ci is the concentration of chlorine at a specific time; Co is the initial concentration of chlorine, which was calculated from the applied amount of calcium hypochlorite that was applied; k is the attenuation rate; t is time (h); and *C*i is the daily average concentration of chlorine.

The R, RSR and IOA values are calculated to assess the reliability and accuracy of the model. The RSR varies from 0 to a large positive value. A lower RSR indicates better simulation performance. The IOA is developed by Willmott^60^ and measures the degree of model simulation error in a range from 0 to 1. When this measure is close to 1, the measurement present high consistency. The detailed calculation formulas for R, RSR and IOA are shown in Equations 23, 24 and 25.

23 $${\rm{R}}=\frac{\sum _{{\rm{i}}=1}^{{\rm{n}}}({{\rm{y}}}_{{\rm{i}}}-\overline{{\rm{y}}})({{\rm{y}}}_{{\rm{i}}}\text{'}-\overline{{\rm{y}}\text{'}})}{\sqrt{\sum _{{\rm{i}}=1}^{{\rm{n}}}{({{\rm{y}}}_{{\rm{i}}}\text{'}-\overline{{\rm{y}}\text{'}})}^{2}\sum _{{\rm{i}}=1}^{{\rm{n}}}{({{\rm{y}}}_{{\rm{i}}}-\overline{{\rm{y}}})}^{2}}}$$

24 $${\rm{RSR}}=\frac{\sqrt{\sum _{{\rm{i}}=1}^{{\rm{n}}}{({{\rm{y}}}_{{\rm{i}}}-{{\rm{y}}}_{{\rm{i}}}\text{'})}^{2}}}{\sqrt{\sum _{{\rm{i}}=1}^{{\rm{n}}}{({{\rm{y}}}_{{\rm{i}}}-\overline{{\rm{y}}})}^{2}}}$$

25 $${\rm{IOA}}=1-\frac{\sum _{{\rm{i}}=1}^{{\rm{n}}}{({{\rm{y}}}_{{\rm{i}}}-{{\rm{y}}}_{{\rm{i}}}\text{'})}^{2}}{\sum _{{\rm{i}}=1}^{{\rm{n}}}{{(y}_{{\rm{i}}}\text{'}-\overline{{\rm{y}}}|+{|y}_{{\rm{i}}}-\overline{{\rm{y}}}|)}^{2}}$$

where **y**_**i**_ and **y**_**i**_′ are the observed and simulated values, respectively, and $\overline{{\bf{y}}}$ and $\overline{{\bf{y}}{\boldsymbol{^{\prime} }}}$ are their average values, respectively.

### Data availability statement

The datasets generated during and/or analyzed during the current study are available from the corresponding author on reasonable request.
